# 

**Published:** 1901-04

**Authors:** 


### CONTENTS.

ORIGINAL COMMUNICATIONS—

Extra-uterine Pregnancy, with Report of a Case of Simultaneous Pregnancy in Both Tubes. By C. R. Robins, M.D., Richmond, Va. .......... 1

Saline Transfusion, Hemorrhage and Shock. By J. L. Campbell, M.D., Atlanta, Ga. .......... 11

SOCIETY REPORTS—

The Clinical Society of Maryland .......... 1[illegible]

EDITORIAL NOTES AND COMMENTS—

A Health Officer for Atlanta .......... [illegible]

NEWS AND NOTES .......... [illegible]

CONTENTS CONTINUED .......... [illegible]

